# Tuberculosis in Pregnant Women After COVID-19: Features of Prevention, Diagnosis, and Treatment (Narrative Review)

**DOI:** 10.3390/jcm14165681

**Published:** 2025-08-11

**Authors:** Anna Starshinova, Ekaterina Belyaeva, Olga Irtyuga, Giunai Sefiyeva, Lubov Mitrofanova, Igor Makarov, Tatiana Makarova, Anastasia Kulpina, Dmitry Kudlay

**Affiliations:** 1 Department of Mathematics and Computer Science, St-Petersburg State University, St. Petersburg 199034, Russia; ekaterina_83@bk.ru (E.B.); asya.starshinova@mail.ru (A.K.); 2 Almazov National Medical Research Centre, St. Petersburg 197341, Russia; irtyuga_ob@almazovcentre.ru (O.I.); namexxx555@mail.ru (G.S.); lubamitr@yandex.ru (L.M.); makarov_ia@almazovcentre.ru (I.M.); t_makarova@mail.ru (T.M.); 3 Department of Pharmacology, Institute of Pharmacy, I.M. Sechenov First Moscow State Medical University, Moscow 119991, Russia; d624254@gmail.com; 4 Institute of Immunology FMBA of Russia, Moscow 115522, Russia; 5 Department of Pharmacognosy and Industrial Pharmacy, Faculty of Fundamental Medicine, Lomonosov Moscow State University, Moscow 119991, Russia

**Keywords:** cardiovascular, COVID-19, multidrug-resistant tuberculosis, pathology, pregnancy, SARS-CoV-2, tuberculosis, treatment

## Abstract

Tuberculosis remains a serious infectious disease that causes over 1.3 million deaths annually. Following the COVID-19 pandemic, the global incidence of tuberculosis has increased to 10.8 million cases. Pregnant women represent a particularly vulnerable population requiring tailored approaches to the prevention, diagnosis, and treatment of tuberculosis. SARS-CoV-2 infection may have impacted existing clinical protocols. Implementing updated methods of tuberculosis prevention, diagnosis, and treatment in pregnant women could help reduce adverse maternal and fetal outcomes. The aim of this review was to explore potential modifications in tuberculosis management among pregnant women in the post-COVID-19 era, including co-infection with SARS-CoV-2. **Methods:** A review was conducted, incorporating a systematic literature search across major international databases, including Medline, PubMed, Web of Science, Scopus, and Google Scholar. The search covered publications released between December 2019 and September 2024 and used targeted keywords such as “COVID-19” OR “SARS-CoV-2”, “tuberculosis” OR “TB” OR “latent tuberculosis infection” OR “pulmonary tuberculosis”, and “pregnancy” OR “pregnant women”. **Results:** Pregnant women living with HIV are at increased risk of developing tuberculosis, which can negatively affect both maternal and perinatal outcomes. Screening for tuberculosis is recommended for all HIV-positive pregnant women, even in the absence of clinical symptoms. Notably, immunological testing before and during pregnancy facilitates the timely and safe detection of tuberculosis infection, enabling preventive and therapeutic interventions during any stage of gestation and the early postpartum period, for the benefit of both mother and child. Drug–drug interactions play a significant role in tuberculosis management, both among anti-tuberculosis agents and with medications for comorbid conditions. Current knowledge of the pharmacokinetics and pharmacodynamics of antituberculosis agents, coupled with therapeutic drug monitoring, supports the development of individualized and effective treatment regimens, which are particularly critical for pregnant patients. Recommendations for managing tuberculosis in pregnant women after COVID-19 infection include measuring D-dimer levels, performing echocardiography, and consulting cardiologists to prevent treatment-related complications. **Conclusions:** Pregnant women represent a distinct subgroup of tuberculosis patients requiring individualized management. Changes observed in tuberculosis progression and treatment responses in pregnant women before and after SARS-CoV-2 infection should inform therapeutic choices, especially in cases of drug-resistant tuberculosis treated with bedaquiline. COVID-19 has been associated with increased cardiovascular risk, which may heighten the likelihood of adverse drug reactions in this population, especially given the limited therapeutic options. Further research is required to assess the long-term outcomes of latent tuberculosis infection in pregnant women and to evaluate the safety and efficacy of novel regimens for drug-resistant TB during pregnancy.

## 1. Introduction

Tuberculosis (TB) remains one of the leading infectious diseases globally, causing more deaths than any other single pathogen. According to the World Health Organization (WHO), approximately 10.8 million new TB cases were reported in 2024, an increase of 3.5% from 10.3 million in 2023. Following the onset of the COVID-19 pandemic, TB incidence increased by 3.9% between 2020 and 2024 [1,2]. In 2022, thirty high-TB-burden countries accounted for 87% of global TB cases, with eight countries comprising two-thirds of all cases: India (27%), Indonesia (10%), China (7.1%), the Philippines (7.0%), Pakistan (5.7%), Nigeria (4.5%), Bangladesh (3.6%), and the Democratic Republic of Congo (3.0%). That same year, 55% of new TB cases occurred in men, 33% in women, and 12% in children (aged 0–14 years) [3]. Pregnant women represent a particularly vulnerable population, especially in high-TB-burden countries [1].

In 2021, the Russian Federation was removed from the list of countries with a high TB burden, but it remained among those with a high burden of multidrug-resistant TB (MDR-TB) and TB/HIV co-infection [1]. In 2022, 16% of all newly detected MDR-TB cases were reported in the WHO European Region [2,4]. In the Russian Federation, 43% of pregnant women diagnosed with TB had MDR-TB, and TB-related mortality among pregnant women with MDR-TB was 8.2% higher than among non-pregnant women [5]. Patients with drug-resistant tuberculosis (MDR-TB) continue to pose one of the greatest challenges globally.

In 2023, an estimated 400,000 people (95% CI: 360,000–440,000) developed multidrug- or rifampicin-resistant TB (MDR/XDR-TB), but only 175,923 were diagnosed and started on treatment, representing fewer than two in five individuals in need and still below pre-pandemic levels (181,533 in 2019) [1,3]. Eighteen countries in Eastern Europe, Central Asia, and the Baltic States accounted for 85% of the global MDR-TB burden. In 2022, MDR-TB prevalence in the region reached 24% among new TB cases and 54% among previously treated patients compared to global rates of 3.3% and 17%, respectively. The incidence of drug-resistant TB remained high, at 2.1 and 21 cases per 100,000 population in these countries [1,3]. As of 2022, the global treatment success rate for MDR-TB was 64%, compared to 55% in Europe and only 51% in the Russian Federation [source: WHO TB Profiles]. The continued spread of MDR-TB is exacerbated by low treatment efficacy in both new and previously treated cases. Since 2019, short-course treatment regimens have been actively developed to enhance therapeutic effectiveness and patient adherence [4].

TB/HIV co-infection remains particularly lethal due to the synergistic effects of immunosuppression and rapid disease progression [5]. Pregnancy adds further complexity, both in diagnostic evaluation and treatment selection, due to the need to minimize risks to the fetus [6,7]. While TB infection can develop at any time during pregnancy, it is more commonly diagnosed in the first half of gestation or within six months postpartum [8]. TB during pregnancy and the first year postpartum tends to follow a more severe course, often involving multi-organ complications [9,10]. Notably, TB with persistent bacterial shedding is more frequently observed in cases developing postpartum than during pregnancy [11,12]. The COVID-19 pandemic has negatively impacted not only global TB incidence and mortality but also the clinical course of TB in pregnant women [13,14]. Many experts express concern that COVID-19 may lead to increased TB incidence in pregnant women due to delayed or missed diagnoses before, during, and after pregnancy [15,16]. Two longstanding challenges have been the limited use of advanced immunodiagnostic methods and restricted access to novel anti-TB drugs in pregnant women, mainly due to the absence of early safety data. SARS-CoV-2 has been shown to significantly affect immunocompromised individuals, leading to post-COVID syndrome and complications in the cardiovascular, endocrine, and nervous systems [17,18,19,20,21,22,23]. These factors may severely restrict the use of anti-TB drugs in pregnant women and increase the risk of adverse drug reactions. The objective of this review is to identify key aspects of TB prevention, diagnosis, and treatment in pregnant women during the post-COVID-19 era, with particular attention to co-infection with SARS-CoV-2.

## 2. Materials and Methods

We conducted a narrative review using a systematic search strategy applied to major international databases, including Medline, PubMed, Web of Science, Scopus, and Google Scholar. The search covered publications published between December 2019 and September 2024, using specific keywords such as “COVID-19” or “SARS-CoV-2”, Post-COVID-19, “tuberculosis” or “TB” OR “latent tuberculosis infection” or “pulmonary tuberculosis”, and “pregnancy” OR “pregnant women”. These keywords were combined using Boolean operators as follows: (“COVID-19” or “SARS-CoV-2”) and (“tuberculosis” or “TB” or “latent tuberculosis infection” or “pulmonary tuberculosis”) and (“pregnancy” or “pregnant women”). For PubMed, the primary search was performed using the official Entrez Programming Utilities (E-Utilities) API, in full compliance with the NCBI Terms and Conditions. The search retrieved 95 articles from PubMed, 47 from Web of Science, 38 from Scopus, 62 from Medline, and 110 from Google Scholar, yielding a total of 352 records. We acknowledge that some articles were duplicated across databases. After removing duplicates, 112 unique articles remained for initial screening. This review was conducted in accordance with the PRISMA 2020 guidelines to ensure methodological rigor and transparency in study selection and data synthesis.

## 3. Background and Post-COVID-19 Maternal Mortality Rates

There is currently no consensus in the scientific literature regarding the impact of asymptomatic or mild COVID-19 on pregnancy outcomes [23,24]. However, no specific morphological alterations distinguishing mild COVID-19 from other acute respiratory infections have been identified [25]. In contrast, severe COVID-19 may significantly worsen pregnancy outcomes by affecting not only the respiratory system but also multiple organ systems. According to the Federal State Statistics Service of the Russian Federation, a sharp rise in maternal mortality per 100,000 live births was recorded during the pandemic, particularly in the second half of 2020 and throughout 2021. The rate, which had been 8.8, 9.1, and 9.0 in 2017, 2018, and 2019, respectively, rose to 11.2 in 2020 and peaked at 34.5 in 2021, before declining to 13.0 in 2022 (*p* < 0.001) (https://rosstat.gov.ru/folder/313/document/137774 accessed on 2 Febrary 2025). Causal analysis indicated that while the distribution of direct obstetric causes remained largely unchanged, indirect obstetric mortality rose significantly in 2020 and 2021, primarily due to severe COVID-19 and its systemic complications. The central mechanism in the pathogenesis of severe COVID-19 is the disruption of host barrier functions. Among the most studied mechanisms is the SARS-CoV-2 infection of endothelial cells and pericytes. This infection causes both direct endothelial damage and indirect effects that impair perfusion, promote thrombosis, and trigger systemic inflammation [26].

Infected pericytes contribute to vascular dysfunction and release mediators that activate endothelial cells in vitro. In later stages, pericyte death and inflammation may result in endothelial barrier breakdown, thrombogenesis, and leukocyte recruitment [27]. Markers of endothelial dysfunction—such as Ang1, VEGF, and VWF—are overexpressed in severe disease [28,29]. In pregnant women, the procoagulant state of pregnancy adds further thrombotic risk in severe COVID-19. Co-infection with active tuberculosis may exacerbate systemic inflammation [22]. The combination of hypercoagulability and inflammation may lead to myocardial ischemia—either type I (thrombotic occlusion) or type II (supply–demand mismatch)—and destabilize hemodynamics in pregnancy.

Although myocarditis has been widely reported in COVID-19, its direct etiologic link remains unconfirmed [30]. Some studies suggest SARS-CoV-2 may reactivate latent enterovirus infections in patients with underlying cardiovascular disease [31]. In pregnancy, this may manifest as peripartum cardiomyopathy, which can progress to end-stage heart failure in severe cases [32]. The subacute phase of COVID-19 may involve myocardial repair and cytokine imbalance. Persistent viral antigens in endothelial cells and pericytes may sustain inflammation [33,34]. Clinically, patients may present with chest pain, arrhythmias, conduction disorders, dyspnea, or reduced exercise tolerance. COVID-19 may also unmask latent cardiovascular conditions—an effect magnified by the physiological cardiac load of pregnancy [35,36].

In addition to its effects on the myocardium and lungs, the vascular-mediated impact of coronavirus infection on the placenta has been extensively documented. SARS-CoV-2 infection during pregnancy significantly increases the risk of placental pathologies such as decidual vasculopathy, fetal vascular malperfusion, maternal and fetal thrombosis, placental infarction, intervillous thrombosis, villitis, and severe placentitis. In many cases, patients also develop acute or chronic histiocytic intervillositis [35,36,37]. These morphological alterations are frequently associated with intrauterine growth restriction and premature rupture of membranes, increasing the risk of adverse perinatal outcomes [38,39].

An additional risk factor for compromised placental perfusion is active tuberculosis. In such cases, non-specific placental abnormalities are observed, including a higher proportion of immature intermediate villi, dystrophic and necrotic lesions, and inflammatory conditions such as villitis and intervillositis. These pathological changes, similar to those in COVID-19, are associated with delayed fetal development [40,41]. From a morphological standpoint, the combination of active COVID-19 and tuberculosis during pregnancy may constitute a significant risk factor for intrauterine growth restriction, due to the synergistic impact of inflammatory, vascular, and structural placental damage.

It is well established that physiological changes during pregnancy are associated with a number of hemodynamic alterations, which are further exacerbated in patients with underlying cardiovascular pathology, thereby affecting both maternal condition and pregnancy outcomes [42]. In this context, the management of pregnant patients with structural cardiac abnormalities and the assessment of risk factors for complications during pregnancy and delivery remain particularly relevant.

Coronavirus disease 2019 (COVID-19) can act as a prothrombotic trigger due to COVID-19-associated endotheliopathy [42], systemic hypercoagulability, and blood stasis resulting from limited mobility. These factors collectively increase the risk of thrombotic complications [43,44].

COVID-19 is associated with hypercoagulability and an increased risk of venous thromboembolism (VTE) as a consequence of systemic inflammation, platelet activation, and endothelial dysfunction [45]. Therefore, it has been hypothesized that since pregnancy itself constitutes a hypercoagulable state and a risk factor for severe COVID-19, pregnant women infected with COVID-19 may have a higher risk of developing VTE compared to their non-infected counterparts. However, this hypothesis has not been consistently supported by existing evidence.

Emily et al. reported that pregnant women with comorbidities such as diabetes mellitus, hypertension, and cardiovascular disease had an elevated risk of severe COVID-19 outcomes, maternal morbidity, and adverse perinatal outcomes [46]. Despite being a high-risk group, pregnant women with these comorbidities should be prioritized for preventive and therapeutic interventions.

In a study by Knight et al. [47], all critically ill pregnant women received prophylactic or therapeutic anticoagulation during hospitalization. Notably, no maternal mortality or cardiomyopathy was observed, and only one case of maternal cardiac arrest occurred, with no increase in VTE incidence among 427 hospitalized pregnant patients with COVID-19.

Similarly, in the U.S., Pierce-Williams et al. [33] found that among 64 hospitalized pregnant patients with COVID-19, 58% received prophylactic and 16% received therapeutic heparin; no VTE cases were reported.

By contrast, another study demonstrated that COVID-19 was associated with a significantly increased incidence of VTE during labor, with a nearly threefold risk compared to non-infected pregnant women, even after adjusting for confounders [48].

COVID-19 has been associated with increased risk of maternal mortality, pre-eclampsia, acute coronary syndrome, arrhythmias, acute kidney injury, and thromboembolism during labor, even after adjustment for pre-eclampsia [49]. Due to its pro-inflammatory nature and severity, COVID-19 may predispose patients to other cardiovascular complications, including myocarditis, heart failure, and arrhythmias [50,51].

While outpatient rates of thrombotic and cardiovascular complications in COVID-19 appear to be relatively low [37,38,39], it remains unclear whether COVID-19 further increases such risks in pregnant outpatients compared to age-matched non-pregnant controls [13,52,53].

To investigate this, the CORONA-VTE-Network registry evaluated cardiovascular and thrombotic events over a 90-day follow-up. Only two thrombotic events occurred in pregnant women, both within 30 days of COVID-19 infection in the third trimester, and no other cardiovascular events were reported [54].

A few additional studies have reported thrombotic and cardiovascular complications in pregnant women with recent COVID-19 [55,56,57]. Collectively, they suggest that pregnant women with COVID-19 are at an elevated risk of cardiovascular complications, particularly VTE. Accordingly, many scientific societies recommend thromboprophylaxis in pregnant and postpartum patients with suspected or confirmed COVID-19, although further clinical validation is necessary [58]. In these patients, therapeutic anticoagulation is essential throughout pregnancy and the postpartum period. The European Society of Cardiology (ESC) recommends individualized anticoagulation regimens based on valve type, position, and risk profile, often involving LMWH or vitamin K antagonists with close monitoring [46,47]. Switching between agents during different trimesters may be necessary, and close collaboration among obstetricians, cardiologists, and hematologists is required [48].

Many pregnant women already receive anticoagulant therapy for various indications. While the safety of prophylactic doses during pregnancy is widely accepted, intermediate and therapeutic doses should be administered under the guidance of a multidisciplinary team, considering gestational age, disease severity, delivery timing, and thrombotic risk [58,59,60,61]. According to the 2021 guidelines of the European Society for Vascular Surgery, low-molecular-weight heparin (LMWH) is the preferred anticoagulant during pregnancy, as it does not cross the placenta or enter breast milk [62].

Denas et al. demonstrated that long-term anticoagulation for various maternal diseases reduces thromboembolic complications and maternal mortality [63]. Another study found benefits from anticoagulation in the acute phase of illness [64]. However, long-term anticoagulation did not reduce morbidity and mortality specifically associated with SARS-CoV-2, highlighting the need for further research [65,66].

Data on intermediate or therapeutic LMWH dosing in pregnancy remain limited. For patients with mild or moderate COVID-19, prophylactic anticoagulation is recommended, despite its limited efficacy in preventing thrombosis and increased bleeding risk. In severe cases, therapeutic LMWH remains beneficial [67].

For patients with prosthetic heart valves, pregnancy management is complicated by hemodynamic abnormalities and physiologic hypercoagulability, posing additional risks of thromboembolic complications (TECs). In addition, the presence of a mechanical prosthetic heart valve increases the risk of bleeding in the case of an emergency delivery for patients taking anticoagulants. In women with biological prosthetic valves, the influence of anticoagulant therapy on pregnancy and fetal outcomes is negated. Although biological prosthetic valves typically do not require anticoagulation, they are still associated with a higher incidence of hemorrhagic and thromboembolic events compared to the general population [68]. In patients with COVID-19 and prosthetic heart valves, antiviral therapy may contribute to unstable INR levels, as shown in studies on non-pregnant populations [69]. These changes in pregnant women with COVID-19 may be particularly important when selecting anti-tuberculosis treatment regimens, especially for drug-resistant TB. Routine D-dimer measurement, echocardiographic monitoring, and cardiologist consultation may be valuable for minimizing complications during therapy.

### Features of the Course of Tuberculosis in Pregnant Women

The coexistence of tuberculosis and pregnancy represents a significant medical and social challenge not only for TB specialists but also for obstetricians, gynecologists, and pediatricians. Key interventions in comprehensive antenatal care should include TB screening as well as the timely treatment and prevention of the disease.

Depending on clinical presentation, pregnant women with active pulmonary TB may be hospitalized at various gestational stages in order to initiate appropriate anti-TB therapy. Although adverse drug reactions occur in approximately 26% of cases, treatment omission poses a far greater risk for maternal and fetal health [70,71]. Delayed treatment or lapses in follow-up are major contributors to morbidity and mortality in both mothers and infants [10]. However, with early treatment initiation and regular follow-up, pregnant women with TB can achieve a cure and favorable maternal outcomes. Importantly, COVID-19 further exacerbates the clinical course of tuberculosis, particularly in cases where SARS-CoV-2 infection is diagnosed prior to or in the absence of anti-TB therapy [72].

Early diagnosis and prompt initiation of anti-TB therapy in pregnant women are crucial for achieving optimal maternal and fetal outcomes. Integrating TB screening into maternal healthcare services—such as antenatal clinics—can enhance case detection, particularly since most TB cases in pregnancy are diagnosed in the third trimester or postpartum period [73,74].

Pregnant women with HIV infection are at heightened risk of TB, which may adversely affect maternal and perinatal outcomes. The WHO recommends TB screening for all HIV-positive pregnant women, even in the absence of clinical symptoms [75]. An understanding of the course of COVID-19 in pregnant women is necessary for characterizing the severity of the specific process and the development of adverse reactions, including those associated with preventive therapy [76].

Post-COVID-19 pulmonary sequelae may obscure clinical symptoms and radiological signs of tuberculosis (TB), complicating the diagnostic process. Residual inflammatory changes, such as fibrosis and infiltrates, necessitate thorough differential diagnosis.

Microscopic examination and culture of sputum remain the gold standards for confirming active TB diagnosis. Novel polymerase chain reaction (PCR) and sequencing techniques can accelerate pathogen identification and detection of drug resistance.

The World Health Organization (WHO) endorses the use of highly sensitive molecular diagnostics, including GeneXpert MTB/RIF and its advanced iterations, which enable rapid detection of *Mycobacterium tuberculosis* and rifampicin resistance. These methods are particularly advantageous in pregnant populations by reducing the need for invasive procedures and expediting treatment initiation.

IGRA tests remain the preferred modality for latent TB infection screening, especially in pregnant women, as they do not require repeat visits for result interpretation, unlike the tuberculin skin test. However, post-COVID-19 immune alterations may affect test sensitivity and specificity, warranting cautious and comprehensive interpretation.

In cases with suspected active TB, chest X-rays with abdominal lead shielding are recommended. Advances in digital radiography have significantly reduced radiation exposure, ensuring safety for both the pregnant patient and the fetus.

Chest CT is reserved for complex cases, particularly when chest radiography is inconclusive or when concomitant pathologies are suspected. The decision to utilize CT imaging should be individualized, weighing gestational age against potential risks.

It is essential to exclude post-COVID-19 pulmonary syndrome, as its clinical and radiological manifestations may overlap with those of TB. A comprehensive diagnostic approach combining clinical evaluation, laboratory testing, and microbiological examination of sputum is required.

## 4. Immunodiagnostic Methods of Tuberculosis Diagnosis for Pregnant Women

Failure to diagnose or treat TB early is associated with a twofold increase in preterm births and a sixfold increase in perinatal morbidity and mortality [4]. Moreover, many TB screening programs were disrupted or reduced during the COVID-19 pandemic [74,75]. Recent advances in immunodiagnostic methods have significantly improved the early detection of tuberculosis infection [76,77].

An early diagnosis of tuberculosis infection is rightfully considered the most important course of action for people infected with tuberculosis mycobacteria, as it facilitates the control of the spread of infection and prevents the development of the disease. Early diagnosis of TB infection is crucial for controlling disease transmission and preventing progression to active TB. Advances in immunodiagnostics—including next-generation assays—have elevated detection capabilities. WHO-endorsed methods include the C-TST, C-Tb, Diaskintest^®^, ELISPOT, QuantiFERON-TB Gold, QuantiFERON-TB Gold Plus, and WANTAI TB-IGRA [6,77,78,79,80].

A new immunological interferon-gamma release assay or IGRA test—TigraTest^®^TB—was registered in the Russian Federation in 2024 (TU 21.20.23-002-89761464-2023 (RU dated 22 April 2024 No.2024/22462)). Although clinical data are still emerging, their use is expanding in routine practice.

Many foreign studies have analyzed the effectiveness of these tests (QuantiFERON-TBPlus, QuantiFERON-TB, and ELISPOT) and compared their significance with that of the Mantoux test with 2TU. Studies, including those conducted by Russian researchers, have demonstrated the high diagnostic value of IGRA tests [56,57], including novel skin tests like C-Tb and Diaskintest^®^ [77,79].

These methods are employed in in vitro TB diagnostics (e.g., ELISPOT), for screening individuals at high risk of TB infection, and for differentiating true TB infection from post-vaccinal hypersensitivity due to prior *M. bovis BCG* vaccination.

In a study analyzing female genital TB, ELISPOT performed on peripheral blood showed a sensitivity of 86% and specificity of 75%, while in peritoneal fluid, these figures rose to 94% sensitivity and 86% specificity [81]. Among women with recurrent miscarriage, IGRA positivity was observed in 28% of cases versus 7% in IGRA-negative controls [82,83]. Across four comparison studies of TST and QuantiFERON-GOLD In-Tube, IGRA tests consistently outperformed TST (diagnostic significance of 91% vs. 77%, respectively) [84,85,86,87].

In 2022, a new ELISA test, WANTAI TB-IGRA, developed in China, was recommended. WANTAI TB-IGRA has characteristics similar to those of QFT-Plus [80]. The sensitivity and specificity of QFT-Plus and TB-IGRA are very similar to those of the WHO-approved IGRA. The results differed for patients with Crohn’s disease (CD), requiring further study.

Russian scientists can rightfully be considered innovators in the development of in vivo tests. In 2008, a group of Russian scientists successfully conducted clinical trials of a new skin test involving the injection of recombinant tuberculosis allergen (Diaskintest^®^). The test uses ESAT-6 and CFP-10 proteins as antigens, which are absent in *M. bovis BCG*, helping to distinguish postvaccine allergies from infectious allergies in 100% of cases [88,89]. The test has been shown to be highly informative in the diagnosis of tuberculosis infection and exhibits performance comparable to that of IGRA tests [87].

Pregnant women constitute a risk group for tuberculosis. The safety and efficacy of the Diaskintest test in determining the activity of tuberculosis infection has been demonstrated in pregnant women previously infected with tuberculosis. Our research team examined 267 pregnant and postpartum women with progressive tuberculosis as well as residual post-tuberculosis changes. All the women underwent a skin test with Diaskintest. A positive result was obtained in 94% of cases for patients with an active specific process. All the women tolerated the Diaskintest skin test well, and there were no negative effects on the fetus in any of the cases. The specificity of this skin test employing a recombinant tuberculosis allergen in active and latent tuberculosis infection was very high. The test is effective and safe for use as a screening test for pregnant women belonging to medical and social tuberculosis risk groups [90].

Notably, according to the literature, the use of immunological tests prior to and during pregnancy enables the timely and safe detection of TB infection and facilitates preventive and therapeutic interventions at any stage of pregnancy or in the early postpartum period, in the interest of maternal and fetal health.

Immunodiagnostics conducted during preconception care allow for the timely exclusion of latent and active TB, thereby increasing the likelihood of favorable outcomes in cases involving assisted reproductive technologies. During consultation, it is necessary to account for a woman’s desire to carry a pregnancy; gestational age; obstetric history (fulfilment of reproductive function); disease activity; the nature, clinical form, and phase of the tuberculosis process; treatment prospects; results of therapy; and tolerance of antibacterial drugs [1,2,6].

## 5. Prevention and Treatment of Tuberculosis in Pregnant Women

The screening and preventive management of TB is considered safe for pregnant women [91,92]. Pregnancy is not a contraindication for treating active TB at any site. Management of TB in pregnancy follows standard therapeutic principles, with additional consideration of potential teratogenic effects, pathogen drug susceptibility, stage of disease, comorbidities, and complications [1,2].

When prescribing therapy, it is paramount to recognize that untreated TB poses a greater risk for both the mother and fetus. Prompt treatment supports favorable pregnancy outcomes and reduces the likelihood of postpartum disease exacerbations [12,13]. As the use of anti-TB drugs during the first trimester may pose a risk to the fetus, it is advisable to postpone initiation of anti-TB therapy until the end of the first trimester in limited forms of tuberculosis without dissemination or bacterial shedding. In cases of widespread, acutely progressive, destructive, and/or complicated forms of tuberculosis, as well as when accompanied by severe comorbidities, treatment should be started immediately after diagnosis, regardless of the patient’s gestational age. For pregnant women infected with HIV, tuberculosis therapy should be started as early as possible due to the elevated risk of disease progression without intervention [3].

Among all TB drugs, only two are classified as safety class B, based on national classification: amoxicillin/clavulanic acid and rifabutin [93]. All other first- and reserve-line drugs are class C and can be used to treat tuberculosis in co-infected pregnant women. The standard regimen (HRZE)—isoniazid (H), rifampicin (R), ethambutol (E), and pyrazinamide (Z)—is recommended during pregnancy (Table 1). These drugs, while classified as category C, have demonstrated safety throughout all trimesters [94].

*Isoniazid (category C)* has no teratogenic effect in animals. Increased hepatotoxicity is possible: during pregnancy and the postpartum period, transaminase activity should be determined monthly. Simultaneous administration of pyridoxine is recommended to prevent neurotoxicity, and vitamin K supplementation is recommended to prevent increased hemorrhage.

Isoniazid tends to inhibit the CYP2E1 isoenzyme of the cytochrome P450 system while it is in the body, with subsequent induction, according to the Compendium of Pharmaceuticals and Specialties [95,96,97]. Because of the inhibition of this isoenzyme, plasma concentrations of iloperidone and acetaminophen, which, in turn, are substrates of CYP2E1, may be altered [98]. In addition to inhibiting CYP, isoniazid acts as a weak inhibitor of monoamine oxidase (iMAO). Clinically significant inter-drug interactions of isoniazid with rifampicin and other strong CYP P450 inducers have been described. In cases where concomitant use of these drugs is required, careful safety monitoring is necessary, including of liver function (especially transaminases like ALT and AST) initially and then every 2-4 weeks during therapy. In addition, isoniazid can inhibit the CYP2C9 isoenzyme, leading to an increase in warfarin concentrations, which may lead to INR lability, an increase in the anticoagulant effect of warfarin, and an increased risk of bleeding [99].

*Rifampicin (category C)*. Being potent inducers of CYP3A4, members of this category can significantly reduce the plasma concentrations of most calcium channel blockers (CCBs) since CYP450 3A4 is the main isoenzyme responsible for their metabolism [100]. Some drug interaction studies have reported significant reductions in the plasma levels of some CCBs, a factor of particular clinical importance in the context of pregnancy therapy, given the high prescription frequency and relatively safe profile of slow calcium channel blockers (SCBs) in the treatment of gestational AH [101,102,103,104]. A dose-dependent increase in the incidence of malformed offspring has been found in animal experiments. Some cases of intrauterine fetal malformations in humans have been reported, but their incidence has not been determined. Drug administration is possible in the second and third trimesters of pregnancy (for vital indications). When administered in the last weeks of pregnancy, rifampicin itself may cause postpartum hemorrhaging in the mother and newborn; vitamin K is prescribed for the prevention of hemorrhagic complications [105].

Rifampicin is also an inducer in the CYP2C9 cytochrome system, where it significantly increases the variability of response to warfarin therapy when co-administered. Rifabutin belongs to the class of rifamycin derivatives; therefore, it also induces the activity of the CYP2C9 isoenzyme, leading to potentially dangerous interactions of the drug with substrates/substrates and concomitant CYP2C9 inhibitors, the most clinically significant of which is mutual interaction with warfarin. With regard to the cytochrome CYP3A4 system, it shows inductor properties similar to those of rifampicin [105,106]. When combined with antacids, the concentration of rifampicin itself may decrease due to increased gastric pH and chelation during oral administration.

*Rifabutin (category C)*. No teratogenic effect has been detected in animal experiments. There are no data on the pharmacokinetics of the drug during pregnancy or clinical experiences of its use in pregnant women. There are insufficient well-controlled study data on the use of amoxicillin or rifabutin in pregnant women with which to discern the risk of the drug. If this drug is used during pregnancy or if a patient becomes pregnant while taking this drug, the patient should be informed of the potential harm to their fetus.

*Pyrazinamide (category C)*. No teratogenic effect was revealed in animal experiments (conducted on mice and rats); however, the data regarding use in pregnant women are limited (albeit several observations of safe use in pregnant women have been published). Animal reproduction studies have shown adverse effects on the fetus, and adequate and well-controlled human studies are lacking, but the potential benefits may justify the use of the drug in pregnant women despite the potential risks. However, if resistance to isoniazid, rifampicin, and ethambutol develops, the use of pyrazinamide in pregnant women may be considered. Its use is recommended in the case of active, destructive tuberculosis.

With regard to pyrazinamide, it should be noted that despite the participation of CYP1A2 and CYP2E1 isoenzymes in its metabolism, a process that leads to the formation of various metabolites, the main pathway of pyrazinamide metabolism is its hydrolysis into pyrazinic acid rather than oxidation, so there are not enough data on clinically significant interactions in the cytochrome P450 system. Paracetamol (acetaminophen), which is considered relatively safe for use during pregnancy at the recommended doses according to FDA updates, may also decrease the rate of pyrazinamide excretion, which may also increase serum levels [107].

*Ethambutol (category C)*. Teratogenic effects have been identified in animal experiments; reports of side effects in pregnant women include a risk of optic neuritis in the child. It should not be prescribed in the first trimester of pregnancy.

Ethambutol has no direct inhibitory effect on CYP activity. Sang Yoon Lee and Himchan Jang et al. determined the inhibitory effect of ethambutol on the activity of nine CYP isoforms—namely, CYP1A2, 2A6, 2B6, 2C8, 2C9, 2C19, 2D6, 2E1, and 3A4—in pooled human liver microsomes (HLMs) [108,109]. The clinical significance of interactions between ethambutol and drugs that are substrates/substrates and inhibitors of the listed cytochrome P450 systems (warfarin, beta-adrenoblockers, calcium channel blockers, proton pump inhibitors, clopidogrel, etc.) within the framework of the variability of response to therapy requires further study (Table 2).

Drugs used for the treatment of drug-resistant tuberculosis—prothionamide (category not defined), ethionamide (category not defined), para-aminosalicylic acid (category C), fluoroquinolones (levofloxacin and moxifloxacin) (category C), cycloserine (category C), capreomycin (category C), linezolid (category C), and delamanid (category C)—are also used during pregnancy, but there is limited evidence regarding their safety. In the Russian Federation (RF), second-line drugs are not recommended for use during pregnancy, alongside optimization of treatment during the ongoing period [3,94] (Table 3).

According to current regulatory documents, the following drugs should not be used in tuberculosis treatment regimens during pregnancy: aminoglycosides, prothionamide (ethionamide), and thioureidoiminomethylpyridinium perchlorate [3].

*Bedaquiline (Category B)*. Bedaquiline (BDQ) has a relatively short history of clinical use for the treatment of MDR-TB, so there are no early indications of the risk it poses to pregnant women. Animal studies have found no evidence of fetal harm. A recent study involving 108 pregnant women and their babies found no safety concerns for women receiving BDQ. The slight decrease in birth weight in the BDQ group could not be attributed to BDQ, with no differences remaining at one year of age. Researchers have found that bedaquiline accumulates significantly in breast milk; breastfed infants receive doses of bedaquiline equivalent to maternal doses [110,111,112,113,114].

Drug interactions have been observed in combinations of bedaquiline and CYP3A4 inducers and inhibitors. When bedaquiline was co-administered with antimycotic drugs from the azole group (fluconazole, voriconazole, posaconazole, etc.), an increase in the concentration of bedaquiline was observed, posing a risk of (potentially severe) adverse reactions; for example, the risk of QTc interval prolongation was multiplied [112].

*Capreomycin (category C)*. Teratogenicity and nephro- and ototoxicity have been detected in animals at high doses. Adequate safety data are not available, and there are no reports of its use in human pregnancy. It can only be used for certain indications.

Capreomycin is not metabolized and is excreted unchanged, mainly by the kidneys. In terms of inter-drug interactions, potentially highly nephrotoxic and ototoxic combinations with vancomycin (category B2) are of clinical significance. It is also dangerous in combination with aminoglycosides (category D) because of a high risk of decreased renal function and neurotoxicity, the mechanism of which is not fully understood but probably involves additive and/or synergistic effects [5]. Paracetamol (acetaminophen) may decrease the rate of capreomycin excretion, leading to an increase in serum levels of capreomycin, with a high likelihood of provoking the development or worsening of adverse reactions to capreomycin [109].

*Levofloxacin and moxifloxacin (category C)*. Fluoroquinolones are not approved for use during pregnancy in young pregnant women (under 18 years of age). Clinical studies of fluoroquinolone use in women of active and no data were obtained on the risk of developing abnormalities in the late reproductive period of pregnancy. Their use for combating MDR and XDR pathogens in pregnant women is especially justified. Administration with or without other drugs is associated with a high risk of arrhythmia due to prolongation of the QTc interval. When these drugs are taken orally, their concentration decreases when co-administered with antacids because of chelation. In combination with rifampicin, a decrease in moxifloxacin concentration mediated by glucuronidation was observed in one study. When taking warfarin, high INR lability can be expected because of the inhibition of warfarin metabolism and intestinal bacteria producing vitamin K [110].

*Cycloserine (category C).* It should be administered with caution during pregnancy and infant feeding, as there are currently insufficient data on the effect of this drug on pregnancy and fetal condition. Cycloserine may exhibit central nervous system (CNS) toxicity (caution is advised when there is evidence of alcohol exposure, a history of seizures, depression, suicidal behavior, or mood instability). In addition, cycloserine may interfere with the absorption of isoniazid. Cycloserine should be used with particular caution for patients with renal impairment and avoided for patients with creatinine clearance <50 mL/min [111].

*Para-aminosalicylic acid (PAS) (category C)*. It should be administered to pregnant women with caution and only for vital indications and in the absence of alternatives for patients with multidrug-resistant tuberculosis. It is worth considering that PAS can reduce rifampicin blood levels—a reason for TLM against rifampicin administration [112].

*Linezolid (category C)*. This drug is used in pregnancy in cases where the expected benefit of therapy for the mother outweighs the potential risk for the fetus. It is unknown whether linezolid is excreted in breast milk; therefore, it is recommended to avoid breastfeeding during therapy with linezolid. This drug is a reversible inhibitor of monoamine oxidases A and B, and serotonin agonists should be administered with caution to avoid serotonin syndrome. In interactions with rifampicin, the plasma concentration of linezolid decreases due to rifampicin’s ability to increase the activity of P glycoprotein.

*Aminoglycosides (kanamycin (category D) and amikacin (category D))* should be avoided during pregnancy [8,12]. Amikacin is excreted by the kidneys and may increase the risk of ototoxicity and nephrotoxicity, especially in cases of prolonged exposure. THM may reduce the risk of adverse events involving aminoglycosides [111]. Drug interactions may be relevant with respect to other drugs exhibiting ototoxicity and nephrotoxicity (e.g., diuretics, cephalosporins, cyclosporine, colistimethate sodium, and tacrolimus).

*Meronem (category C)*. Safety in pregnancy has not been studied. Experimental studies on animals have not shown any adverse effects on the developing fetus. The only adverse event identified in animal studies on the effect of the drug on the reproductive system was an increased abortion rate in monkeys when administered at a dose 13 times higher than the recommended dose for humans. Meronem should not be administered to pregnant individuals unless the potential benefit of its use justifies the possible risk to the fetus. In each case, the pre-medication should be used under the direct supervision of a physician [3]. This drug has less convulsant activity than ABPs of the same group of carbapenems—imipenem/cilastatin. But co-administration of these antibiotics significantly reduces the concentration of valproic acid (VA) in serum to subtherapeutic levels immediately after the start of co-administration and consequently increases the risk of seizures [113,114]. Seizures have also been reported after the administration of carbapenems alone without VC (according to the WHO). Patients with central nervous system disorders (organic brain lesions, a history of seizures, and bacterial meningitis) are at high risk.

*Delamanid (category C).* This drug is contraindicated during pregnancy and breastfeeding (as it exerts teratogenic effects, with high concentrations in breast milk). All pregnant women should have easy access to information on TB drug therapy. However, as pregnant women are frequently excluded from TB research, they are often overlooked [115,116]. No obvious teratogenic effect has been identified, but fetal delay has been noted following administration of the drug at high doses. It is contraindicated in the first trimester of pregnancy [117]. When co-administered with strong inducers of CYP3A4 enzymes (e.g., rifampicin), its concentration is markedly decreased. Caution is recommended when this drug is administered alongside clofazimine [117,118] and fluoroquinolones due to the additive risk of QTc interval prolongation and the development of fatal arrhythmias.

*Clofazimine (category C).* Animal studies have shown some adverse effects. No human clinical trials have been conducted. The benefit of the drug is greater than the possible risk during pregnancy [119,120]. The drug should be avoided in the first trimester of pregnancy. It can be used during pregnancy according to strict indications. In addition, in vitro studies have determined the inhibitory activity of clofazimine against CYP2C8 and CYP2D6 isoenzymes. Clofazimine can prolong the QTc interval; therefore, interaction with class IA (quinidine and novocainamide) and class III (amiodarone and sotalol) antiarrhythmic agents can prolong the QTc interval through additive effects [121].

*Aminoglycosides (streptomycin, amikacin, and kanamycin).* These should not be used during pregnancy due to their teratogenicity (safety class D).

*Kanamycin (category D)*. Teratogenicity has not been demonstrated in animal studies. By penetrating through the hematoplacental barrier (like other aminoglycosides), it may cause damage to the fetus. There are data indicating the drug has an embryotoxic effect: in high doses, it may cause irreversible dystrophy of the auditory nerve in the fetus, resulting in the development of congenital deafness (ototoxic effect); in this case, we are referring to both the administration of the drug to a pregnant woman and the occurrence of pregnancy against the backdrop of its administration.

*Terizidone (category D).* This drug is teratogenic and fetotoxic in animals. Its use is contraindicated during pregnancy. There are no controlled studies of its use in a human pregnancy. It can only be applied for vital indications. Terizidone, consisting of two molecules of cycloserine, has the same pharmacological properties as cycloserine but can be administered to patients with creatinine clearance <50 mL/min and patients on dialysis with appropriate dose adjustment. Terizidone exhibits lower CNS penetration and generally has better tolerability.

*Pretomanid (category D)*. This drug should be used during pregnancy only if the benefit to the patient is considered to outweigh the potential risk to the fetus. It has proven teratogenic effects and embryotoxicity (involving the development of skeletal anomalies and fetal growth retardation). It is not known whether pretomanid/metabolites are excreted in human breast milk. Available pharmacodynamic/toxicological data pertaining to animals have shown excretion of pretomanid with milk. A risk to the infant cannot be excluded. The decision to discontinue breastfeeding or pretomanid therapy should be made in consideration of the benefits of breastfeeding for the child and the benefits of therapy for the woman [3].

According *to in vitro* studies, CYP3A4 is responsible for a 20% contribution to the metabolism of pretomanid.; Therefore, co-administration of pretomanid with strong or moderate inducers of CYP450 3A4 may reduce the plasma concentration and antimicrobial effect of pretomanid. As shown in the literature, after seven days of concomitant administration, rifampicin (600 mg daily) reduced the systemic exposure (AUC) and peak plasma concentration (Cmax) of pretomanid by 66% and 53%, respectively. Dexamethasone can also significantly reduce the plasma concentration of pretomanid [111,112].

*Prothionamide, Ethionamide (category D)*. Animal studies have revealed teratogenicity (CNS and skeletal malformations). Its use is contraindicated during pregnancy. There are no adequate studies on human pregnancy. Increased nausea and vomiting have been noted. It should only be used when indicated [3].

Thus, the critical role of potential drug–drug interactions in tuberculosis (TB) treatment is evident, both within combinations of anti-TB drugs and in regimens that include medications for concomitant comorbidities. This underscores the vital importance of therapeutic drug monitoring (TDM), which facilitates achieving optimal blood concentrations of anti-TB drugs, thereby enhancing treatment efficacy and minimizing adverse effects. A thorough understanding of the pharmacokinetic and pharmacodynamic properties of antituberculosis agents, when integrated with TDM, provides an essential framework for tailoring individualized and effective therapeutic regimens—an aspect of particular significance in pregnant patients.

In cases of severe tuberculosis, cardiopulmonary insufficiency, or severe concomitant extragenital pathology (e.g., decompensated bronchial asthma, chronic obstructive pulmonary disease, diabetes mellitus, chronic kidney disease, cardiovascular disorders), initiation of antituberculosis therapy is recommended irrespective of gestational age, prioritizing the preservation of maternal and fetal life [122,123].

Due to the high risk of developing tuberculosis in pregnant women, preventive measures are most important in this risk group.

## 6. Vaccinations Against COVID-19 in Pregnant Tuberculosis Patients

Accumulating clinical evidence and real-world data support the safety of SARS-CoV-2 vaccines—including Sputnik V and mRNA-based vaccines (Pfizer-BioNTech, Moderna (USA, New York City)—in pregnant women. A meta-analysis encompassing 71 studies, involving 17,719,495 pregnant women and 389 pregnant animals, demonstrated no significant association between vaccination and adverse pregnancy outcomes across vaccine types or trimesters. Most included studies (94%) were conducted in high-income countries, with cohort designs predominating (51%), and 15% assessed as having a high risk of bias [124,125]. The sole notable exception was an increased incidence of postpartum hemorrhage (10.40%; 95% CI: 6.49–15.10%) reported following COVID-19 vaccination in two studies [125].

Despite these reassuring findings, further safety investigations are warranted for non-mRNA-based COVID-19 vaccines. Data from the Russian Ministry of Health indicate the safety of Sputnik V (Gam-COVID-Vac) in pregnant women, despite their initial exclusion from vaccination guidelines due to limited information. In a cohort of 9667 pregnant women vaccinated with Gam-COVID-VAC, no serious adverse events were documented. Nonetheless, additional comprehensive studies are necessary to elucidate vaccine safety during pregnancy and to develop targeted organizational strategies aimed at improving vaccination uptake during pregnancy planning.

Pregnant women with chronic diseases (obesity, diabetes, cardiovascular disease, etc.) are at high risk of developing severe COVID-19. For this population, vaccination is particularly recommended from the second trimester onwards (from 22 weeks). Vaccination is also recommended for breastfeeding women, as the data do not indicate risks for infants. There are currently no direct data available on interactions between COVID-19 and TB vaccines in pregnant women.

Before the vaccination of a pregnant woman with tuberculosis, a comprehensive examination should be carried out, including an assessment of the activity of the infectious process and immune status. It is possible to assume activation of the immune response and stimulation of active specific inflammation against the background of tuberculosis infection. At the same time, it is necessary to consider the change in immune status against the background of pregnancy. This issue requires further investigation and determination of the risk for the woman and her fetus, including the risk posed by acute and chronic infectious processes.

Thus, the forms of managing pregnant tuberculosis patients with and without COVID-19 have some differences pertaining to the development of viral infection. We have presented the differences in Table 4.

The presented tactics for examination and management of pregnant women at risk of developing tuberculosis infection are consistent with current WHO recommendations [4,7,93]. Following these recommendations will reduce the incidence of tuberculosis in pregnant women, especially in the post-COVID-19 period.

## 7. The Combination of COVID-19 and Tuberculosis in Pregnant Women

A 26% increase in childhood TB cases in the EU/EEA in 2023 suggests ongoing community transmission, which could indirectly affect pregnant women due to household exposure [1,126]. The combination of COVID-19 and tuberculosis in pregnant women poses a serious medical challenge, as both diseases can exacerbate each other and increase the risk of complications for the mother and fetus. Key aspects of this co-infection include the risk of severe COVID-19 in pregnant women with tuberculosis, the impact of infection on the fetus, diagnostic challenges, and simultaneous treatment of both infections [126].

Pregnant women with tuberculosis are at high risk of severe COVID-19 due to the physiological decline in immunity during pregnancy. Simultaneously, it is necessary to consider the impact of COVID-19 on the reduction in immune response, which, in combination with tuberculosis, may act as a dual risk factor [127]. Pregnant women with latent TB infection (LTBI) may face an increased risk of TB reactivation if exposed to COVID-19, due to immune modulation during pregnancy. It is also important to consider the dual impact of both infections on lung tissue, which can lead to respiratory failure and other complications such as pneumonia or acute respiratory distress syndrome (ARDS). Severe COVID-19 (especially with the Delta variant) increases the likelihood of ARDS, thrombosis, and mortality. Meanwhile, tuberculosis increases the incidence of anemia (24%), gestosis (18%), and placental insufficiency (20%) [128,129]. Caesarean section (48–100% of cases) is indicated in severe TB or COVID-19 with respiratory failure [126].

Pregnant women with tuberculosis and COVID-19 have an increased risk of premature birth, fetal growth restriction, and placental insufficiency. Premature birth occurs in up to 25% of cases, as well as fetal growth restriction and low birth weight [130,131]. Tuberculosis intoxication and inflammatory processes in COVID-19 can exacerbate anemia and gestosis in pregnant women. In addition, vertical transmission of tuberculosis to the fetus is possible, but rare. This mainly occurs when the placenta is affected. According to current data, COVID-19 is not transmitted in utero, but a newborn can become infected after birth.

There are also difficulties in diagnosing two infectious processes. The symptoms of tuberculosis and COVID-19 are similar (cough, fever, weakness), which complicates differential diagnosis or exacerbates the course of tuberculosis [127].

X-ray examinations, which are absolutely necessary for the diagnosis of tuberculosis, should be performed with caution in pregnant women. However, if active tuberculosis or severe COVID-19 is suspected, this examination method is mandatory, taking into account the stage of pregnancy [128,129,130].

**Figure 1:**
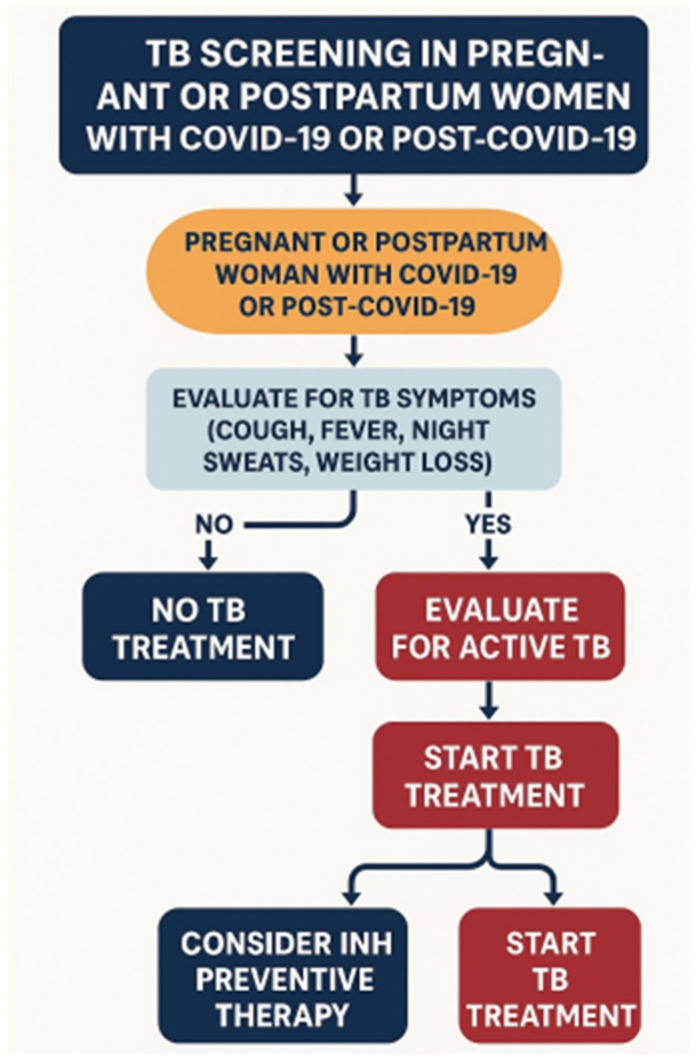
The algorithm for TB diagnosis in pregnant or postpartum women with COVID-19 and post-COVID-19.

After all necessary measures have been taken, pregnant women with tuberculosis should begin or continue anti-tuberculosis therapy. In cases of COVID-19 requiring additional therapy, especially immunosuppressive therapy, anti-tuberculosis therapy is of significant importance. Remdesivir, dexamethasone (in severe cases), and anticoagulants (heparin) are used. Monoclonal antibodies and molnupiravir are being studied, but there is insufficient data on their safety for pregnant women [44,45,46,131]. Supportive therapy is used to treat COVID-19 in pregnant women, and in severe cases, oxygen support and antiviral drugs are used. The changes described in pregnant women during and after COVID-19 infection may be particularly important when selecting anti-tuberculosis therapy, especially when treating drug-resistant tuberculosis with bedaquiline [132]. During and after COVID-19, there is an increase in the incidence of cardiovascular disease, which may increase the number of adverse events, with a limited selection of drugs available for use. Further knowledge is needed on the long-term follow-up of pregnant women with latent infection, as well as on the efficacy and safety of new treatment regimens for drug-resistant tuberculosis during pregnancy. Vaccination against COVID-19 is recommended from the second trimester of pregnancy. The necessary algorithm for TB diagnosis in pregnant or postpartum women with COVID-19 and post-COVID-19 is presented in Figure 1.

Thus, the combination of COVID-19 and tuberculosis in pregnant women requires a specialized multidisciplinary approach. Timely prevention, diagnosis, and adequate treatment of infections minimise risks to the mother and child.

The diagnosis of tuberculosis (TB) in pregnant women presents unique clinical challenges, further complicated by the ongoing effects of the COVID-19 pandemic. Post-COVID-19 immune dysregulation may increase susceptibility to both primary TB infection and reactivation of latent tuberculosis infection (LTBI), particularly in high-burden settings. The World Health Organization (WHO) and the U.S. Centers for Disease Control and Prevention (CDC) provide guidance on TB screening and diagnosis in pregnancy, with growing attention to post-COVID considerations. Similar efforts are being made in the Russian Federation to implement new guidelines for the diagnosis of tuberculosis in pregnant women.

According to WHO guidelines (2022), all pregnant women with symptoms of TB—such as persistent cough, fever, night sweats, and weight loss—should undergo clinical screening, regardless of COVID-19 history (WHO, 2021) [4,77,94]. However, in the post-COVID-19 context, there is heightened emphasis on systematic screening among women with previous SARS-CoV-2 infection, particularly those with underlying immunosuppressive conditions (e.g., HIV, diabetes, or malnutrition), given the potential for increased TB risk after COVID-19-induced immune compromise (WHO, 2023;) [4]. Current WHO guidance highlights the importance of considering recent COVID-19 as a potential confounder in TB diagnosis, due to overlapping respiratory symptoms and radiologic features. Clinical judgement is essential to distinguish post-COVID lung damage from active TB, especially in the presence of fibrotic changes or persistent cough. In such cases, bacteriological confirmation becomes especially important to avoid misdiagnosis.

## 8. Conclusions

Tuberculosis in pregnant women remains an important challenge from detection to treatment. COVID-19 has significantly complicated the course of many diseases, including tuberculosis [127,128,129]. Analyses of the literature have shown that the number of publications, including our own studies, on COVID-19 and tuberculosis in pregnant women is extremely limited. Nonetheless, in high-TB-burden countries, immunological diagnostic methods for tuberculosis infection are essential for early detection of the disease. Currently, no significant changes have been introduced in the principles of tuberculosis diagnostics. However, more careful risk assessment of tuberculosis development in pregnant women who have had COVID-19 is necessary due to the higher risk of tuberculosis activation.

Existing immunodiagnostic methods are feasible for use, with high efficacy and safety in pregnant women. The possibility of cardiological complications in the context of COVID-19 requires a more detailed examination of pregnant women when tuberculosis infection is detected, and there is a need to prescribe anti-tuberculosis therapy [131,132,133,134]. The described changes in pregnant women during and after COVID-19 infection may be of particular relevance in the selection of anti-tuberculosis therapies, especially in the treatment of drug-resistant tuberculosis. D-dimer determination, echocardiography, and cardiology consultation for preventing further adverse events and complications during therapy may be important recommendations for pregnant women. The presence of immunosuppression against the backdrop of COVID-19 infection, especially during pregnancy, may reduce the effectiveness of immunodiagnosis via skin tests. Suspicion of the development of COVID-19-related pneumonia requires verification via CT, which is associated with increased radiation exposure, but is necessary to determine how to manage the patient. The changes described for pregnant women before and after COVID-19 infection may be of particular importance in the selection of antituberculosis therapies, especially in the treatment of drug-resistant tuberculosis with bedaquiline. Particularly necessary is the careful selection of therapy in the event of the detection of drug-resistant tuberculosis. Bedaquiline is currently the main drug included in the corresponding therapy regimen. Increased cardiovascular morbidity has been observed during and after COVID-19 infection, which may increase the number of adverse events for which there is a limited range of possible drugs available for treatment.

There is a need for further accumulation of knowledge on long-term follow-ups of pregnant women with latent infection, as well as on the efficacy and safety of new regimens for the treatment of drug-resistant tuberculosis in pregnancy.

## Figures and Tables

**Table 1:** Mechanisms of action and key interactions of anti-tuberculosis drugs.

TB Drug	Pregnancy Category	Mechanism of Action/ Metabolism	Pathogenesis	Interaction with Drugs	Risk of Adverse Reactions
Isoniazid	C	Antibacterial; inhibits mycolic acid synthesis	Inhibits then induces CYP2E1; inhibits CYP2C9; weak MAO inhibitor	Increases warfarin levels; interactions with rifampicin	Hepatotoxicity risk; monthly liver enzyme monitoring; supplement with vitamins B6 and K
Rifampicin	C	Bacterial RNA polymerase inhibitor	Potent inducer of CYP3A4, CYP2C9	Decreases plasma levels of calcium channel blockers, warfarin, some antibiotics	Contraindicated in 1st trimester; risk of bleeding in mother and fetus; vitamin K prophylaxis recommended
Pyrazinamide	C	Hydrolyzed to pyrazinoic acid	Metabolism involving CYP1A2, CYP2E1 (minor role)	Possible reduced clearance with aspirin, paracetamol	Limited data; use if benefits outweigh risks
Ethambutol	C	Antibacterial	Inhibits CYP1A2, CYP2E1; moderate inhibition of CYP2C19, CYP2D6; weak inhibition of CYP3A4 and others	Possible interactions with warfarin, beta-blockers	Risk of optic neuropathy; contraindicated in 1st trimester
Rifabutin	C	CYP3A4 and CYP2C9 inducer	Similar to rifampicin	Alters warfarin and other CYP3A4 substrate levels	Insufficient data; potential risks
Levofloxacin	C	Inhibits bacterial DNA gyrase	Partially metabolized via glucuronidation	Reduced levels with rifampicin; interacts with warfarin	Used in MDR-TB; risk of QT prolongation
Moxifloxacin	C	Inhibits bacterial DNA gyrase	Similar to levofloxacin	Same as levofloxacin	Same as levofloxacin
Bedaquiline	B	Inhibits mycobacterial ATP synthase	Metabolized by CYP3A4	Interaction with azoles (increased levels), rifampicin (decreased levels)	Requires QT monitoring; passes into breast milk
Capreomycin	C	Aminoglycoside antibiotic	Not metabolized by CYP	Increased nephro- and ototoxicity with other drugs	High toxicity; caution in pregnancy
Cycloserine	C	Cell wall synthesis inhibitor	No CYP interactions	Interactions with isoniazid, CNS drugs	Risk of neurotoxicity; use with caution
Para-aminosalicylic acid	C	Antibiotic	May reduce rifampicin levels	May decrease rifampicin efficacy	Use only if no alternatives
Linezolid	C	Protein synthesis inhibitor	Unknown CYP effects	Interacts with serotonergic drugs, rifampicin	Not recommended during breastfeeding
Amikacin/Kanamycin	D	Aminoglycosides	Not metabolized by CYP	Increased nephro- and ototoxicity	Contraindicated due to fetal hearing loss risk
Meropenem	C	Carbapenem; cell wall synthesis inhibitor	No direct CYP effects	Lowers valproic acid levels; risk of seizures	Use cautiously, especially with CNS pathology
Delamanid	C	Inhibits mycobacterial respiration	Metabolized by CYP3A4	Reduced efficacy with rifampicin; increases QT interval	Contraindicated in pregnancy and breastfeeding
Clofazimine	C	Membrane stabilizer; antibacterial	Inhibits CYP3A4, CYP2C8, CYP2D6	Risk of QT prolongation; interactions with antiarrhythmics	Use only if strictly indicated
Thioacetazone	D	Cycloserine derivative	Unknown CYP effects	Toxic	Contraindicated in pregnancy

**Table 2:** Data on drugs used to treat tuberculosis, along with the drug sensitivity of the pathogen.

Anti-Tuberculosis Drugs	Experimental Data	The General Clinical Symptoms
Isoniazid	It has no teratogenic effect on animals.	Hepatitis: Nausea, vomiting, right upper quadrant abdominal pain, jaundice, elevated ALT/AST/bilirubin. Risk increases with age, chronic liver disease, and alcohol abuse. Peripheral neuropathy: Paresthesia (numbness, tingling), pain in hands/feet. CNS: Dizziness, headache, rarely seizures or psychosis. Neuropathy is associated with vitamin B6 (pyridoxine) deficiency. Prevented by B6 supplementation.
Rifampicin	In animal experiments, a dose-dependent increase in the incidence of malformed offspring has been found. Some cases of intrauterine fetal malformations in humans have been reported, but their incidence has not been determined.	Hepatitis: See Isoniazid. Frequently occurs in combination with H. Colors bodily fluids (urine, saliva, tears) orange-red. May stain contact lenses. GI tract: Nausea, vomiting, diarrhea, abdominal pain. Immune system: Flu-like syndrome with fever, chills, headache, myalgia, arthralgia (more common with intermittent dosing). Thrombocytopenia, hemolytic anemia.
Rifabutin	No teratogenic effect was detected in animal experiments.	
Pyrazinamide	No teratogenic effect was revealed in animal experiments (conducted on mice and rats); however, the data on its use in pregnant women are limited.	Hepatitis: See Isoniazid. Frequently increases uric acid levels. Jhyperuricemia, gouty arthritis: Joint pain, swelling, redness (often first metatarsophalangeal joint). GI tract: Nausea, vomiting, anorexia. Metabolism: Asymptomatic ↑ uric acid.
Ethambutol	Teratogenic effects have been identified in animal experiments; reports of side effects in pregnant women include a risk of optic neuritis development in the child.	Retrobulbar neuritis: ↓ visual acuity, ↓ red-green color discrimination, scotomas, eye pain. Key toxicity! Dose-dependent (risk ↑ at >15 mg/kg/day). Requires ophthalmologic monitoring. Nervous system: Peripheral neuropathy (less common than with H).

↑—high level;↓—low level.

**Table 3:** Significant data on drugs used to treat multidrug-resistant tuberculosis.

Anti-Tuberculosis Drugs	Experimental Data	Risks of Using the Drug	The General Clinical Symptoms
Bedaquiline	Animal studies have found no evidence of fetal harm.	There are no early indications of its risk to pregnant women.	QT prolongation: Risk of ventricular arrhythmias. ECG monitoring is mandatory. Liver: ↑ ALT/AST (rarely severe hepatitis). GI tract: Nausea, abdominal pain
Capreomycin	Teratogenicity and nephro- and ototoxicity have been detected in animals at high doses.	It can only be used when indicated.	Vestibular: Dizziness, imbalance, nystagmus. Risk ↑ with high doses, prolonged use, renal impairment, and advanced age. Requires audiometry. Nephrotoxicity: ↑ creatinine/urea, proteinuria, rarely acute renal failure. Neuromuscular blockade (especially with rapid IV administration or in myasthenia): muscle weakness, respiratory depression.
Levofloxacin and moxifloxacin	Against the background of high doses of levofloxacin, delayed bone development in the fetus was noted in preclinical studies. An increased incidence of spontaneous abortions and increased postnatal mortality were also observed.	The use of fluoroquinolones is not approved for young pregnant women (under 18 years of age). Clinical studies of fluoroquinolones in pregnant women of active and late reproductive age have not shown a risk of fetal anomalies.	Nausea, vomiting, diarrhea, abdominal pain. QT interval: Mfx > Lfx. Risk ↑ with hypokalemia, QT-prolonging drugs, heart disease. CNS: Headache, dizziness, insomnia, rarely seizures or psychosis. QT prolongation: Risk of ventricular arrhythmias (torsades de pointes). Tendinitis, tendon rupture (especially Achilles). Risk ↑ with age, corticosteroids, renal impairment.
Cycloserine	There are not enough data on the effect of this drug on pregnancy and fetal condition.	This drug should be administered with caution during pregnancy and infant feeding	Depression, anxiety, psychosis, suicidal ideation, confusion, headache, seizures, tremor. Dose adjustment or discontinuation often necessary.
Para-aminosalicylic acid	No teratogenic effect has been revealed in animal experiments (mice and rats); however, reports of its administration to pregnant women are limited.	It should be administered to pregnant women with caution, only for vital indications and in the absence of alternatives in patients with multidrug-resistant tuberculosis.	Nausea, vomiting, diarrhea, abdominal pain. Hepatitis: See Isoniazid. Hypothyroidism: (with prolonged use), goiter. Allergic reactions: Rash, fever, rarely Stevens–Johnson syndrome.
Linezolid	No teratogenic effect has been observed in animal experiments (mice and rats); however, there are few reports of its administration to pregnant women.	This drug is administered to pregnant women in cases when the expected benefit of therapy for the mother outweighs the potential risk for the fetus.	Optic neuropathy: ↓ vision. Neuropathy more common with prolonged use. GI tract: Nausea, diarrhea. Serotonin syndrome: (especially with SSRIs): Agitation, hallucinations, hyperthermia, tachycardia, hyperreflexia, myoclonus. Thrombocytopenia: Bleeding, petechiae. Anemia: Fatigue, pallor. Leukopenia/neutropenia: Infection risk. Dose- and duration-dependent. Requires CBC monitoring. Risk ↑ if used >28 days. Peripheral neuropathy: Paresthesia, pain (often irreversible).
Clofazimine	Animal studies have shown some adverse effects. No human clinical trials have been conducted.	The drug should be avoided in the first trimester of pregnancy. It can be used during pregnancy according to strict indications.	Reddish-brown pigmentation (especially exposed areas). Dry skin, ichthyosis, itching. Pigmentation usually reversible after discontinuation, but fades slowly. GI tract: Abdominal pain, nausea, diarrhea. Corneal crystal deposition (usually asymptomatic), dry eyes.
Delamanid	No obvious teratogenic effect has been identified, but fetal delay has been noted with high doses of the drug.	This drug is contraindicated in the first trimester of pregnancy.	QT interval prolongation. Increases the risk of ventricular arrhythmias, especially when combined with other QT-prolonging agents (e.g., bedaquiline, fluoroquinolones). Regular ECG monitoring is required. Nausea, vomiting, dyspepsia, abdominal pain. Usually mild and transient. Rarely progresses to clinical hepatitis. Hypokalemia, hypomagnesemia, hypoalbuminemia. Electrolyte disturbances may increase the risk of QT prolongation.
Prothionamide and Ethionamide	Animal studies have revealed teratogenicity (CNS and skeletal malformations).	Its use is contraindicated during pregnancy.	Nausea, vomiting, anorexia, metallic taste, abdominal pain. Taking with food or at bedtime may reduce side effects. Hepatitis: See Isoniazid. Hypothyroidism: (with prolonged use), goiter. Peripheral neuropathy, depression, rarely psychosis.
Pretomanid	This drug has proven teratogenic effects and embryotoxicity (namely, the development of skeletal development anomalies and fetal growth retardation).	It should be used during pregnancy only if the benefit to the patient outweighs the potential risk to the fetus.	Gastrointestinal (GI): Nausea, vomiting, diarrhea, anorexia. Common. May contribute to weight loss. Hepatotoxicity is a significant concern, particularly when combined with bedaquiline and linezolid (e.g., BPaL regimen). Central Nervous System (CNS): Headache, dizziness, insomnia. Peripheral neuropathy: Risk increases in combination with linezolid. Myelosuppression (especially when used with linezolid). Requires regular complete blood count (CBC) monitoring. Rash, pruritus. Rarely severe (e.g., Stevens–Johnson syndrome).

↑—high level; ↓—low level.

**Table 4:** The management of pregnant women with tuberculosis with and without COVID-19.

Measures	Pregnant Women with Tuberculosis	Pregnant Women with Tuberculosis and COVID-19
Assessment of clinical symptoms	There are usually no clinical manifestations.	Appearance of a fever and cough.
The radiological complex of the examination and	X-ray.	CT of the chest is preferred.
Immunodiagnostics of tuberculosis infection	Immunodiagnostics of tuberculosis infection (IGRA tests, Diaskintest) are safe.	The sensitivity of skin tests may be reduced against the background of immunosuppression in COVID-19.
Laboratory tests	Microscopy and sputum culture for *Mycobacterium tuberculosis* (Mtb).	Microscopy, sputum culture for *Mycobacterium tuberculosis* (Mtb); PCR for SARS-CoV-2; Leucocytosis and thrombocytopenia are presented, which should be monitored.
Treatment	Treatment of tuberculosis based on drug susceptibility.	TB treatment and treatment of COVID-19 (remdesivir, dexamethasone, and anticoagulans). Monoclonal antibodies and molnupiravir are being studied, but safety data for pregnant women are insufficient.
Delivery and postnatal period	No premature delivery is indicated.	Caesarean section is indicated in cases of severe TB combined with COVID-19 when respiratory failure develops.
Prevention	Vaccination against COVID-19 (mRNA vaccine) from the second trimester onwards.	Co-administration by a phthisiatrician, obstetrician–gynecologist, and infectious-disease specialist.

## Data Availability

No new data were created or analyzed in this study.
